# Biochar-Immobilized *Pseudomonas aeruginosa* Enhances Copper Remediation and Growth of Chinese Milk Vetch (*Astragalus sinicus*)

**DOI:** 10.3390/microorganisms13081793

**Published:** 2025-07-31

**Authors:** Yunkai Hu, Chuan Wang, Youbao Wang

**Affiliations:** 1 School of Ecology and Environment, Anhui Normal University, Wuhu 241002, China; 2 Fuyang Center for Disease Control and Prevention, Fuyang 236000, China

**Keywords:** *Pseudomonas aeruginosa*, biochar immobilization, copper remediation, heavy metal soil, Chinese milk vetch, nitrogen fixation, plant growth promotion

## Abstract

Heavy metal-contaminated soil poses a severe threat to environmental quality and human health, calling for eco-friendly and efficient remediation strategies. This study explored the use of biochar-immobilized copper-resistant *Pseudomonas aeruginosa* to remediate copper-contaminated soil and promote growth of Chinese milk vetch (*Astragalus sinicus* L.). Indoor pot experiments compared four groups: copper-contaminated soil (control), soil with biochar, soil with free bacteria, and soil with biochar-immobilized bacteria (IM). Results showed IM had the most significant effects on soil properties: it raised pH to 7.04, reduced bioavailable copper by 34.37%, and increased catalase (3.48%) and urease (78.95%) activities. IM also altered soil bacterial communities, decreasing their richness and evenness (alpha diversity) while shifting community composition. For Chinese milk vetch, IM reduced leaf malondialdehyde (a marker of oxidative stress) by 15%, increased total dry weight by 90%, and lowered copper accumulation in roots (18.62%) and shoots (60.33%). As a nitrogen-fixing plant, the vetch’s nitrogen fixation in roots and shoots rose by 82.70% and 57.08%, respectively, under IM. These findings demonstrate that biochar-immobilized *Pseudomonas aeruginosa* is a promising in situ amendment for remediating copper-contaminated soil and boosting plant growth.

## 1. Introduction

Considering the development and expansion of industrial production, unreasonable waste discharge, and biogeochemical processes, the heavy metal content in soil in some areas has seriously exceeded the standard, resulting in pollution. This condition directly or indirectly threatens environmental quality and human health and urgently needs to be properly treated. Soil heavy metal pollution, particularly copper (Cu) contamination, arises from anthropogenic and natural processes. Copper is an essential trace element for the growth of animals and plants, but it also has biological toxicity [1]. The copper in soil does not degrade, and its accumulation causes the destruction of soil structure, deterioration of soil physical and chemical properties, nutrient imbalance, downregulation of microbial activity, and reduction of crop yield [2,3]. Finally, copper-contaminated soil affects peoples’ health through the food chain [4,5].

These harms necessitate efficient, eco-friendly remediation strategies to mitigate Cu bioavailability and restore soil function. Conventional remediation technologies for heavy-metal-polluted soil focus on either removing metals or immobilizing them in situ. While physical/chemical methods exist, their high cost and environmental disruption drive interest in sustainable bioremediation. Biochar and microbial remediation offer promise but face limitations when applied alone.

Biochar is an organic material made from agricultural wastes by high-temperature combustion in an anoxic or anaerobic environment [6]. The raw materials for biochar preparation are widely sourced, and the cost is relatively low. Biochar has a unique structure with many pores, a large specific surface area, many negative charges on the surface, and rich functional groups containing oxygen, nitrogen, and sulfur [7]. It can increase the cation exchange capacity and pH of soil and is currently widely applied in the field of environmental pollution control [8,9,10]. However, in the application process of biochar, some problems such as the reduction in soil fertility [11,12], the decrease in crop production [13,14], the release of heavy metals previously adsorbed into the environment again [15,16], and the secondary pollution caused by modified biochar [17] might be encountered.

Microbial remediation is a green and efficient means of environmental remediation. Bacteria can directly control the behavior of metals through the accumulation of metals in cells or the adsorption on the cell surface, and they can also convert or detoxify metals through enzymatic hydrolysis and redox reactions [18]. Nevertheless, free bacteria are vulnerable to environmental stressors (e.g., pH shifts and competition), limiting field efficacy. To overcome these constraints, immobilized microbial technology (IMT) synergizes both approaches. Immobilized microbial technology (IMT) refers to a biotechnology that could fix and enrich free microorganisms to an area by physical or chemical methods [19]. This technology overcomes the limitations of planktonic cells (e.g., low stability, poor reusability, and susceptibility to environmental stress) by leveraging carriers or matrices to enhance microbial retention and functionality. Considering the unique porous structure, biochar can be taken as an ideal carrier of exogenous microorganisms in soil for IMT [20]. Beyond controlling organic pollutants like petroleum hydrocarbons [21], IMT has broadened its scope to heavy metal remediation, degradation of xenobiotics, and wastewater treatment [22]. Moreover, immobilized microorganisms can also stabilize soil heavy metals, improve soil enzymatic activities, and ameliorate soil properties [23,24]. Among various immobilization microbial technologies, the nano-immobilization microbial system stands out particularly. It provides a multi-functional platform for environmental remediation, featuring high stability, continuous activity, and strong interaction capabilities with pollutants. In terms of stability, nanocarriers can better protect microorganisms from external environmental disturbances, maintaining the integrity of their structure and function. In terms of interaction with pollutants, nanomaterials’ high surface area and adsorption capacity concentrate pollutants (e.g., heavy metals, dyes) around immobilized microbes, accelerating degradation [25]. Moreover, nano-immobilized microbes exhibit prolonged activity due to reduced cell washout and protected enzyme stability [25]. Based on these advantages, using nano-immobilized microorganisms for environmental remediation has become an important research direction at present. The promoting effect of immobilized microorganisms on plant growth and phytoremediation has been widely reported in recent years. This finding explains the role of immobilized microorganisms in soil remediation from another perspective. Ref. [26] studied the ability of biochar-immobilized cadmium-resistant bacteria to promote the efficiency of cadmium phytoextraction by *Chlorophytum laxum* R.Br. Ref. [27] found that the inoculation of *Bacillus* in association with biochar sp. was beneficial for soil and plant growth amelioration and the microbial community activity.

Notably, the interactions between Cu and microbial systems are deeply influenced by the redox properties and coordination chemistry of Cu, as highlighted by studies on dinuclear Cu(II) complexes [28]. These complexes exhibit tunable biological activities—including catecholase, antimicrobial, and antibiofilm effects—mediated by the redox behavior of Cu(II)/Cu(I) couples and the structure of coordinating ligands (e.g., pyrazine derivatives and methoxybenzoates). Specifically, ligand substituents can modulate the electron density of Cu centers, altering their reactivity toward biomolecules and microbial cells. For instance, dinuclear Cu(II) complexes with 3-methoxybenzoate ligands show enhanced catecholase activity due to increased redox accessibility of Cu(II), facilitating electron transfer during substrate oxidation [28]. Such mechanisms parallel the interactions between Cu-resistant microbes like *Pseudomonas aeruginosa* and environmental Cu: microbial enzymes (e.g., reductases, oxidases) may exploit Cu redox transitions to detoxify or sequester Cu, while microbial metabolites could act as endogenous ligands to stabilize Cu in less bioavailable forms.

In the present study, straw biochar was used to immobilize a copper-resistant strain (*Pseudomonas aeruginosa*) selected from the farmland soil of tailings. Chinese milk vetch (*Astragalus sinicus* L.), which is the main winter green manure crop in the southern rice region of China, was planted in copper-contaminated soil with different treatments. The effects of the immobilized bacteria on the remediation of copper-contaminated soil and the growth of Chinese milk vetch had been fully analyzed to provide reference for the bioremediation of heavy-metal-polluted soil.

## 2. Materials and Methods

### 2.1. Materials

The bacterial strain was a copper-resistant strain screened from the farmland soil of Laoyaling tailings in Tongling City, Anhui Province, China (117°53′12.84″ E, 30°54′6.48″ N). The 16S rDNA sequence of the copper-resistant strain *Pseudomonas aeruginosa* (strain K1) was submitted to the GenBank database, and the homology analysis was performed using BlastN (http://blast.ncbi.nlm.nih.gov/, accessed on 22 April 2022). A phylogenetic tree was constructed based on the 16S rDNA sequence comparison to identify the strain. This identification method is consistent with the approach described in the study by [29], where the 16S rDNA sequencing and BlastN homology analysis were used to confirm that strain K1 isolated from the farmland soil of Laoyaling tailings belongs to *Pseudomonas aeruginosa*. Its minimum inhibitory concentration (MIC) in copper was 996.7 mg·L^−1^. The optimum growth conditions of the strain were pH 7 and 30 °C.

The biochar was purchased from a company in Zhenjiang City, Jiangsu Province, China. It was prepared from mixed straw of wheat and rice (1:1) at 600 °C under limiting oxygen conditions, with a yield of approximately 30%. The obtained biochar was crushed and sieved through a 100-mesh sieve to measure its physical and chemical properties. The pH value of the biochar was determined to be 8.7 by using a PHS-3C pH meter (Youke, Shanghai, China). The peak particle size and specific surface area of the biochar were 44.25 μm and 166.9 m^2^·kg^−1^, respectively, as determined using a Bettersize2000E laser particle size distribution analyzer (Better, Dandong, China).

For the preparation of the immobilized bacteria, a purified single colony of the copper-resistant strain was inoculated into Luria–Bertani (LB) medium for activation at 30 °C and 130 rpm by using a SHZ-A water-bath thermostatic oscillator (Boxun, Shanghai, China) for 24 h. The bacterial culture was inoculated on an immobilized medium with biochar at a ratio of 2% and cultured at 30 °C and 130 rpm for 48 h to obtain immobilized bacteria by centrifugation [30].

The seeds of Chinese milk vetch were purchased from Zhengzhou Kaiyuan Grass Science and Technology Co., Ltd. (Zhengzhou, China). The soil samples were collected from the 10–20 cm soil layer at the peach garden on the Huajin campus of Anhui Normal University. Tree branches, stones, and other impurities were removed before the soil was passed through a 2 mm sieve for further use. The 10 g·L^−1^ copper stock solution prepared using CuSO_4_·5H_2_O was added to the soil, mixed well, and aged for 30 days to simulate 400 mg·kg^−1^ copper-contaminated soil as the remediation object.

### 2.2. Experimental Design

Indoor pot experiments were conducted with the four following treatments:

Copper-contaminated soil (CK): the copper concentration of soil was 400 mg·kg^−1^.

Copper-contaminated soil + biochar (BC): the proportion of biochar was 0.2% (2 g·kg^−1^).

Copper-contaminated soil + free bacteria (FB): the bacteria were cultured in 100 mL of LB medium at 30 °C and 130 rpm for 48 h, centrifuged at 5000 rpm for 10 min, resuspended with 50 mL of deionized water, and applied to 500 g of soil.

Copper-contaminated soil + immobilized bacteria (IM): The immobilized bacteria were cultured in 100 mL of immobilized medium (with 1 g biochar) at 30 °C and 130 rpm for 48 h, centrifuged at 5000 rpm for 10 min, resuspended with 50 mL of deionized water, and applied to 500 g of soil.

The above soils were divided into 12.5 cm diameter pots. Each pot was filled with 500 g of soil. Four pots were used for each treatment, including three for planting and one for control. In the planting group, 30 seeds of Chinese milk vetch were sown per pot. All the pots were placed in a greenhouse with 14 h·day^−1^ artificial light and irrigated with deionized water every morning to adjust the soil moisture to 60–70%. A tray was installed at the bottom of the pot to avoid copper loss with water. The planting period lasted for 4 months.

### 2.3. Methods

#### 2.3.1. Scanning Electron Microscopic (SEM) Observation

The free or immobilized bacteria were rinsed with 0.9% normal saline 2–3 times, fixed with 2.5% glutaraldehyde, and dehydrated with a gradient of 20%, 50%, 80%, and 100% ethanol. Finally, the ethanol was replaced with acetone and stored at 4 °C. The bacterial morphology and surface structure of the biochar were observed using JSM-6390LV SEM (JEOL, Tokyo, Japan).

#### 2.3.2. Determination of Soil Properties

With reference to the national standard method (HJ 962-2018), the pH of soil was determined using the PHS-3C pH meter (Youke, Shanghai, China). With reference to the national standard method (HJ 802-2016), the electrical conductivity (EC) of soil was determined using a DDS-11A digital conductivity meter (Youke, Shanghai, China). The organic matter (OM) in soil was determined by potassium dichromate colorimetry by using a 752 UV–vis spectrophotometer (Jinghua, Shanghai, China).

With reference to the national standard method (HJ/T 166-2004), the available copper in soil was extracted with diethylene triamine pentaacetic acid and determined using a 4510 flame atomic absorption spectrophotometer (FAAS, Jingke, Shanghai, China). The decline rate of available copper was calculated using Equation (1).

(1) $$R\left(\%\right)=\frac{C_{0}-C_{t}}{C_{0}}\times 100\%$$

where *R* is the removal rate (%), *C*_0_ is the initial concentration (mg·L^−1^), and *C_t_* is the residual concentration (mg·L^−1^).

Based on the methods of [31], the catalase activity was determined by potassium permanganate titration and expressed in milliliters of 0.1 mol·L^−1^ KMnO_4_ consumed by 1 g of air-dried soil cultured for 20 min. Urease activity was determined by phenol sodium colorimetry, and the results were expressed in milligrams of NH_3_-N in 1 g of air-dried soil cultured at 37 °C for 24 h.

#### 2.3.3. Analyses of Soil Bacterial Community

Microbial DNA was extracted from soil samples by using the E.Z.N.A.^®^ soil DNA kit (Omega Bio-tek, Norcross, GA, USA). The V4–V5 region of the bacteria 16S ribosomal RNA gene was amplified by polymerase chain reaction (PCR) at 95 °C for 2 min, followed by 27 cycles at 95 °C for 30 s, 55 °C for 30 s, and 72 °C for 60 s. A final extension was carried out at 72 °C for 5 min. The primers were 515F 5′-barcode-GTGCCAGCMGCCGCGG-3′ and 907R 5′-CCGTCAATTCMTTTRAGTTT-3′. Amplicons were extracted from 2% agarose gels and purified using the AxyPrep DNA gel extraction kit (Axygen Biosciences, Union City, CA, USA). Purified PCR products were quantified using Qubit^®^3.0 (Life Invitrogen, Carlsbad, CA, USA), and every 24 amplicons whose barcodes were different were mixed equally. The pooled DNA product was used to construct an Illumina Pair-End library based on Illumina’s genomic DNA library preparation procedure. The amplicon library was paired-end sequenced (2 × 250) on an Illumina MiSeq platform (BIOZERON, Shanghai, China).

#### 2.3.4. Indicators of Chinese Milk Vetch

Malondialdehyde (MDA) in leaves was extracted with trichloroacetic acid and determined by UV–vis spectroscopy. Plant samples of Chinese milk vetch were carefully removed from the soil after the whole planting and then rinsed with deionized water. The roots and shoots of the plants were separated and oven-dried at 75 °C for 24 h to measure the dry weight. The dried root and shoot samples were acid-digested to determine the copper and nitrogen concentrations in plants by FAAS.

### 2.4. Statistical Analyses

The data were expressed as means and standard errors from the triplicate values. One-way ANOVA and multiple comparisons were analyzed at 95% confidence by using SPSS 20.0. The data and graphs were processed using Microsoft Office Excel 2016.

Sequences were clustered into operational taxonomic units at 100% similarity by using the Deblur denoising algorithm [32]. The phylogenetic affiliation of each 16S rRNA gene sequence was analyzed using the uclust algorithm against silva (SSU138.1). The 16S rRNA database was used at a confidence threshold of 80% [33].

Rarefaction analysis based on Mothur v.1.21.1 was conducted to reveal the alpha diversity indexes [34]. Beta diversity analysis was performed using UniFrac to compare the results of the principal component analysis (PCA) by using the community ecology package [35]. All statistical analyses of sequencing data were performed using the R stats package (R-4.1.0).

## 3. Results

### 3.1. SEM Observation

**Figure 1:**
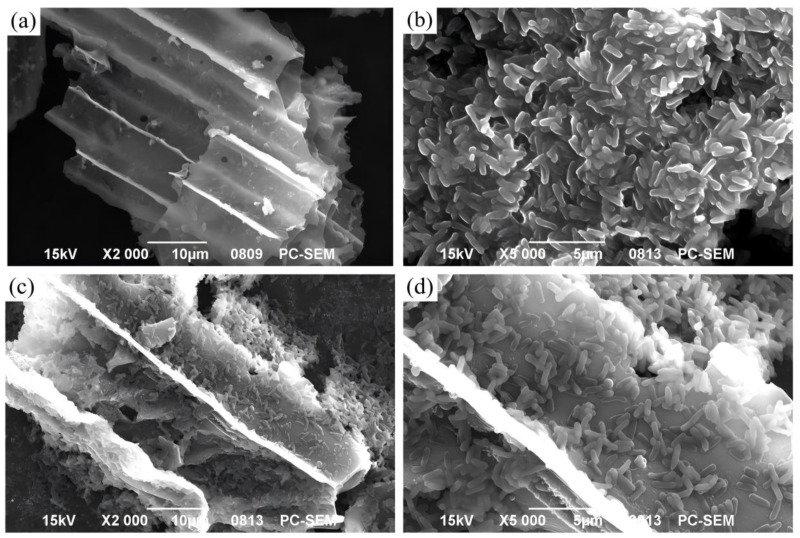
SEM image of immobilized bacteria. (**a**) Morphology of biochar under SEM at 2000× magnification. (**b**) Morphology of *Pseudomonas aeruginosa* under SEM at 5000× magnification. (**c**) Morphology of immobilized bacteria under SEM at 2000× magnification. (**d**) Morphology of immobilized bacteria under SEM at 5000× magnification.

Under the SEM, the straw biochar had a porous tubular structure (Figure 1a). *Pseudomonas aeruginosa* presented oval and short rods with a smooth surface (Figure 1b). A large number of immobilized bacteria attached to the surface of biochar, and the morphology was consistent with that of *P. aeruginosa* (Figure 1c,d). This finding indicates that the copper-resistant strain was successfully immobilized on the biochar.

### 3.2. Effects of Different Treatments on Soil

**Figure 2:**
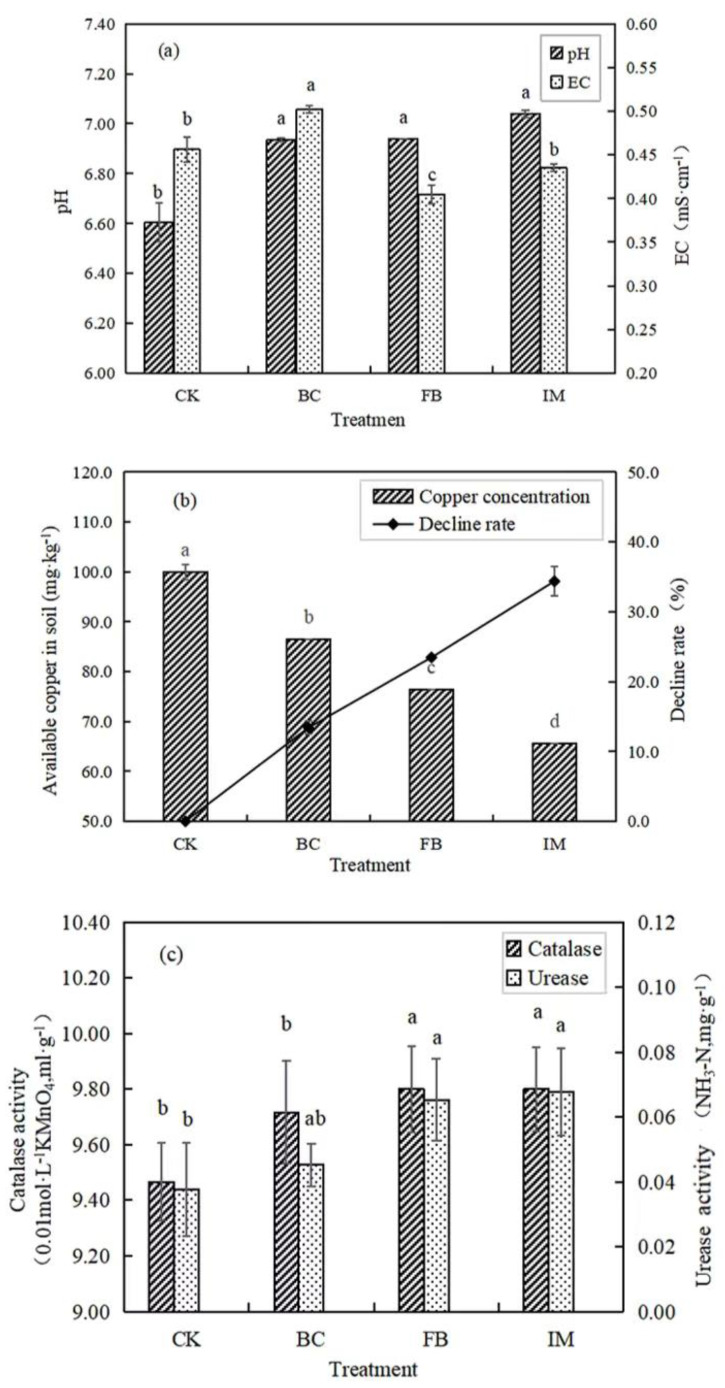
Effects of different treatments on EC and pH of soil (**a**), available copper content in soil (**b**), and soil enzymatic activities (**c**). Different lowercase letters after the data indicate a significant difference between treatments (*p* < 0.05).

The soil pH values of BC, FB, and IM were significantly (*p* < 0.05) higher than that of CK. Among all the treatments, IM had the largest increase in pH, from 6.61 to 7.04. In comparison with CK, the soil EC (Electrical Conductivity) of BC increased from 0.456 to 0.502, and this condition might be related to the large amount of charge on the surface of biochar. FB treatment significantly (*p* < 0.05) reduced the EC of soil to 0.405, while IM reduced it to 0.435 (*p* > 0.05, Figure 2a).

The soil available copper content of CK was 99.95 mg·kg^−1^, which was significantly (*p* < 0.05) decreased by the treatment with BC, FB, and IM to 86.55, 76.50, and 65.60 mg·kg^−1^, respectively. The decline rates in BC, FB, and IM were 13.41%, 23.46%, and 34.37%, respectively. Hence, IM had the best stabilization effect on copper in soil (Figure 2b).

The experimental results showed that both FB and IM treatments significantly (*p* < 0.05) increased the activities of catalase and urease, while the increase induced by BC was not significant (*p* > 0.05). Among all treatments, the soil enzyme activities of IM were the highest. In comparison with CK, the catalase activity of IM increased by 3.48%, and the urease activity increased by 78.95% (Figure 2c).

### 3.3. Effects of Different Treatments on the Composition of the Soil Bacterial Community

The sequencing coverage of all the treatment groups was higher than 97.7%, suggesting that the sequencing depth was sufficient to meet the subsequent analysis requirement. By ranking the treatments in accordance with the Shannon index and evenness, the orders were both the same as IM < FB < CK < BC (*p* < 0.05). Based on the Simpson index, the order was BC = FB < CK < IM (Table 1). Clearly, the treatment IM could reduce the alpha diversity of soil bacteria, while BC could improve it.

**Figure 3:**
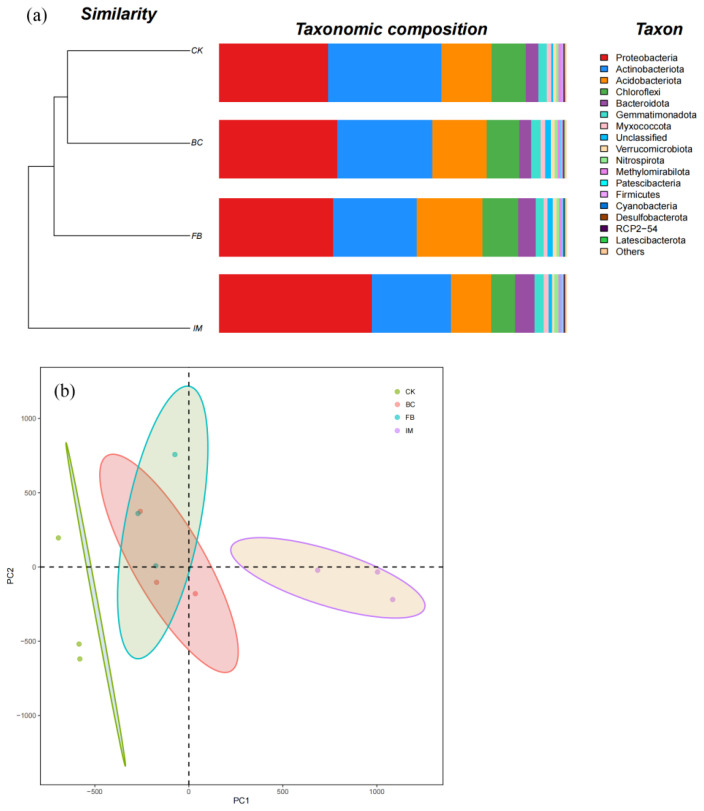
Soil bacterial community. (**a**) Composition and treebar of bacterial community at the phylum level. Different species are represented by the columns of different colors, and the relative abundance of species is represented by the length of columns. (**b**) Principal component analysis (PCA). Different treatments are represented by points of different colors, and the confidence ellipses describe the confidence region of scatter points.

As shown in Figure 3a, the top six phyla in relative abundance were *Proteobacteria*, *Actinobacteriota*, *Acidobacteriota*, *Chloroflexi*, *Bacteroidota*, and *Gemmatimonadota*. The phylum with the highest relative abundance (32.62%) in CK was *Actinobacteriota*. The relative abundance of *Actinobacteriota* decreased when the soils were treated with BC, FB, and IM, whereas that of *Proteobacteria* increased. The phylum with the highest relative abundance (43.92%) was *Proteobacteria* in IM. This finding indicated that IM treatment could change the dominant bacterial species in soil. Based on the cluster tree, CK and BC were clustered together, followed by FB and then IM (Figure 3a). The PCA results also indicate that the compositions of BC and FB were close to each other between CK and IM. The composition of IM was obviously different from that of CK (Figure 3b).

### 3.4. Effects of Different Treatments on Chinese Milk Vetch

**Figure 4:**
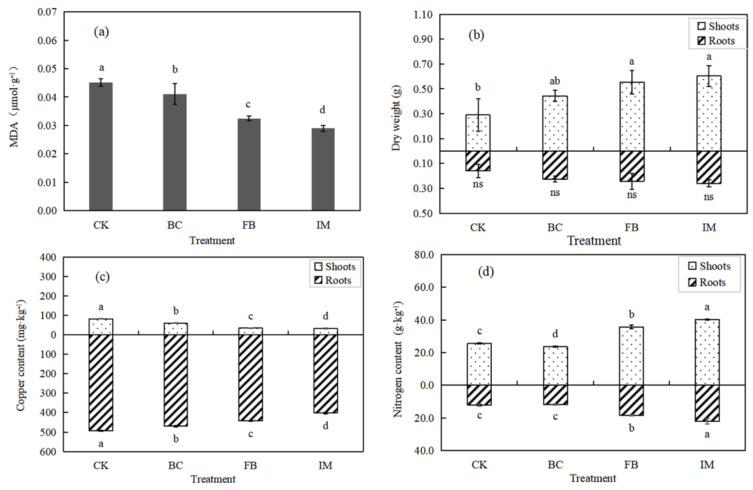
Effect of treatments on Chinese milk vetch. (**a**) MDA content in leaves. (**b**) Dry weight of roots and shoots. (**c**) Copper content in shoots and roots. (**d**) Nitrogen content in shoots and roots. Different lowercase letters indicate a significant difference between treatments (*p* < 0.05), and “ns” indicates no significance (*p* > 0.05).

The MDA contents in the leaves of Chinese milk vetch were in the order of CK > BC > FB > IM (*p* < 0.05), with the highest decrease rate of 35.56% in IM, indicating a reduced stress on leaves (Figure 4a). By contrast, the biomass of Chinese milk vetch showed an increasing trend of CK < BC < FB < IM. The promoting effect of treatments on dry weight was evident (*p* < 0.05) in the shoots, while that in the roots was not significant (*p* > 0.05). The dry weight of the whole plant in IM was the greatest and was 91.78% higher than that of CK (Figure 4b).

Figure 4c suggests that the copper bioaccumulation of Chinese milk vetch showed a significant (*p* < 0.05) decrease by the treatments both in shoots and roots. This finding is consistent with the change in available copper content in soil and opposite to the change in plant biomass. Moreover, the copper contents in roots were much higher than those in shoots with a very low translocation factor. The copper content in roots decreased from 493.583 mg·kg^−1^ (CK) to 401.667 mg·kg^−1^ (IM), corresponding to 18.62%. The copper content in shoots decreased from 81.500 mg·kg^−1^ (CK) to 32.333 mg·kg^−1^ (IM), corresponding to 60.33%.

The nitrogen amounts of Chinese milk vetch significantly (*p* < 0.05) increased in FB and IM and decreased in BC. Different from the distribution of copper in plants, the nitrogen contents in shoots were higher than those in roots. The highest nitrogen contents were observed in IM, in which the values increased by 82.70% in roots and 57.08% in shoots (Figure 4d).

## 4. Discussion

### 4.1. Biochar Colonization of Immobilized P. aeruginosa

*P. aeruginosa* was successfully colonized on the biochar. The process of microorganism attachment depends on the material properties of biochar, such as the surface roughness, topography, free energy, surface charge, and hydrophobicity [36]. Especially, biochar has an important characteristic, which is its porous structure [10]. The porous structure can serve as habitats for soil bacteria or fungi [37].

Ref. [38] found that the strains of *Leocobacter* sp. and *Bacillus aryabhattai* cultivated on the solid medium in the presence of biochar formed colonies, produced exopolysaccharides, and formed biofilms. However, the extracts of the same biochar suppressed their growth. Thus, the structure, composition, and surface functional groups of biochar can affect the colonization by microorganisms. For instance, the low-molecular-weight hydrocarbons on biochar surfaces may be utilized by microorganisms as a carbon source [39]. They can also stimulate signal molecules or inhibit microbial activity and plant growth [40].

### 4.2. Remediation of Copper-Contaminated Soil by Immobilized P. aeruginosa

The results suggest that both biochar and *P. aeruginosa* could be used as repair agents of polluted soil, and the remediation effects of immobilized bacteria on copper-contaminated soil were the best. Although biochar can directly stabilize heavy metals through ion exchange, electrostatic interaction, physical adsorption, complexation, and precipitation, its capacity is limited. Once the adsorption of heavy metal ions reaches the limit, pollutants may be released to the environment again [15,16]. Excessive application of biochar may also have adverse effects on soil quality [41,42]. Therefore, biochar is not an ideal soil conditioner.

Heavy-metal-resistant strains can remove heavy metal ions from the environment through extracellular complexation, precipitation, adsorption, and fixation, as well as transformation, absorption, and utilization in vivo [43]. When microorganisms are adsorbed onto the solid surface, the surface charges, the number of active sites, and the potential heavy metal adsorption capacity of microorganisms and carriers will be improved [44,45,46]. When biochar is combined with resistant bacteria as an immobilized carrier, it can help the bacteria resist environmental interference and stress, promote the growth and enrichment of bacteria, and achieve the sustainability of environmental remediation through the continuous passage of microorganisms [20,47,48,49].

The immobilized *P. aeruginosa* could increase the pH of soil, because the pH value of biochar is 8–9. When it is applied to the soil, the biochar will increase the soil pH [50,51,52,53]. Another reason is that *P. aeruginosa* can increase the pH of the environment through metabolism [54]. Ref. [55] found that the pH of culture medium inoculated with *P. aeruginosa* could rise to 8.74 during the growth process. The ability of immobilized *P. aeruginosa* to increase environmental pH may enhance its tolerance to heavy metal stress in soils.

The toxicity and bioavailability of heavy metals in soil mainly depend on their available state, that is, the water-soluble and exchangeable heavy metals that are easily absorbed and utilized by plants. Given the increase in pH induced by immobilized *P. aeruginosa*, the negative charge in the soil increased, and heavy metal ions in the soil were converted from soluble to insoluble, coupled with the adsorption of heavy metals by biochar and bacteria, leading to the decrease in available copper and EC in soil. The results are consistent with those of [24,30], indicating the stabilization of heavy metals in soil by immobilized bacteria.

Soil enzymatic activities can reflect the activation of soil microorganisms, and they are also important indicators of soil quality and soil pollution [49,56]. The immobilized *P. aeruginosa* could also improve soil enzymatic activities, consistent with the research results of [30,57]. Three factors may influence this property. First, the effect of immobilized bacteria on soil pH can cause it to deviate from or approach the optimal pH of soil enzymes to affect the enzymatic activities. Moreover, biochar improved the growth and metabolism of microorganisms by providing breeding sites and nutrients for them in order to increase the content of enzymes in soil [58,59]. Third, the decrease in the concentration of available copper in soil reduced the microbial growth inhibition in order to make microorganisms secrete more enzymes [60].

Accordingly, biochar-immobilized *P. aeruginosa* could be used as an in situ remediation agent in copper-contaminated soil.

### 4.3. Changes in the Composition of Soil Bacterial Community

The alpha diversity indexes decreased the most because of the immobilized *P. aeruginosa* treatment, including the Shannon index and evenness. The Simpson index was the opposite because of its reciprocal form. Based on the relative abundance, the dominant phylum of soil bacteria changed from *Actinobacteriota* to *Proteobacteria* by the application of immobilized *P. aeruginosa*.

According to the systematical evolution relationship, *P. aeruginosa* belongs to *Proteobacteria*, which might be the most metal-tolerant organisms found at metal-contaminated sites [61,62]. In immobilized *P. aeruginosa*, the porous structure and large specific surface area of biochar can promote the growth of microorganisms by providing protection space and boosting the soil aeration and water retention [63,64,65]. In addition, the biochar could enhance the competitiveness of *P. aeruginosa* loaded on it with the other bacteria in the soil, thus inducing the disappearance of some species with low abundance or competitiveness [6,27]. As a result of its copper resistance and the sheltering effect of biochar, *P. aeruginosa* has become a dominant species in copper-contaminated soil through extensive reproduction and reduced the diversity and evenness of soil bacteria. The diversity of soil bacteria increased when biochar was applied alone, because biochar provided a carrier and space for the growth of all soil microorganisms aside from *P. aeruginosa.* The clustering tree and PCA both revealed that biochar, *P. aeruginosa*, and immobilized *P. aeruginosa* significantly altered the composition of the soil bacterial community, with the latter having the most remarkable effect. This result is consistent with the findings of [27], thus confirming the above judgment.

### 4.4. Effects of Immobilized P. aeruginosa on the Growth of Chinese Milk Vetch

Chinese milk vetch contains certain ornamental and economic value as an organic feed or nectar source plant. The indicators of Chinese milk vetch were analyzed to further investigate the remediation effect of immobilized *P. aeruginosa* on copper-contaminated soil.

Excessive exposure to heavy metals can induce oxidative stress, leading to reactive oxygen species, such as hydroxyl radical (OH), which will undergo peroxidation with fatty acids in the cell membrane, producing MDA [66]. Thus, the content of MDA indirectly reflects the degree of damage to the membrane system and the stress resistance of plants [67]. Both biochar and heavy-metal-tolerant bacteria strains could reduce the MDA content of plants [68,69]. Similarly, biochar, *P. aeruginosa*, and immobilized *P. aeruginosa* decreased the MDA of Chinese milk vetch in the present study, with the latter having the most pronounced effect. This finding indicates that the treatments can reduce the growth stress of copper in the soil on the plants, which may be related to the decline of available copper content in soil.

The excessive accumulation of MDA may threaten the survival of plants by affecting the metabolic pathway [16,70,71,72]. Therefore, the MDA content and biomass of plants are often inversely proportional. The results of the present study indicate that the biomass of Chinese milk vetch was increased by the soil treatments, and the growth-promoting effect of immobilized *P. aeruginosa* was the most significant. Biochar can improve crop yield by decreasing bulk density, increasing soil porosity, bettering water and nutrient status, and strengthening soil aeration [73,74]. However, the excessive application of biochar could cause high soil alkalinity, resulting in a deficiency of plant micronutrients. Ref. [12] found that 15% biochar application caused retardation of plant growth compared with the control group. By contrast, 5% and 10% biochar promoted plant height and fresh weights of shoot and root. Hence, the proportion (0.2%) of biochar in the present study was set to a very low value. The biochar was only used as a carrier for bacteria without adverse effects on soil and crops. *P. aeruginosa* is a kind of plant-growth-promoting rhizobacteria (PGPR) [75,76,77,78]. Ref. [67] reported that the inoculation of *P. aeruginosa* and *Burkholderia gladioli* increased the biomass of tomato (*Solanum lycopersicum*) under Cd stress, probably because the bacteria could induce the upregulation of the plant defense system, heavy metal tolerance proteins, and phytohormones [79].

The trend of the copper concentration of Chinese milk vetch was consistent with the changes in the available copper content in soil and MDA content in leaves but opposite to the dry weight of plants. The trace elements in plants mainly come from the soil. Hence, the decrease in available copper content in soil directly leads to a decrease in copper absorption by plants. The roots are the first to come into contact with copper stress, comprising a major process in metal absorption and translocation [69]. Thus, the copper concentrations in the roots of Chinese milk vetch were higher than those in the shoots, which were close to or even higher than the copper concentration in the soil. The growth dilution effect of plants can also lead to a decrease in the concentration of heavy metal elements in them [80,81]. Consequently, Chinese milk vetch planted in the soil treated with immobilized *P. aeruginosa* contained the lowest amount of copper both in the roots and shoots.

The strong nitrogen fixation capacity and high utilization efficiency of Chinese milk vetch allow the stimulation of a large amount of nitrogen and improvement of soil fertility during plant decomposition, which plays an important role in maintaining the nitrogen cycle in farmland ecosystems [82]. The current experimental results show that treatments with *P. aeruginosa* and immobilized *P. aeruginosa* enhanced the nitrogen fixation ability of Chinese milk vetch and induced stronger nitrogen transport capacity. Immobilized *P. aeruginosa* has the greatest promoting effect, which has a positive significance in promoting the growth of Chinese milk vetch and improving farmland fertility. However, the treatment with biochar did not have this effect.

## 5. Conclusions

A copper-resistant *P. aeruginosa* screened from the farmland soil of Laoyaling tailings in Tongling was inoculated with straw biochar to develop an environmentally friendly and efficient bioremediation material for heavy-metal-contaminated soil. Biochar provided a habitat and nutrition for the growth and reproduction of *P. aeruginosa*, thereby immobilizing the bacteria on its surface. When immobilized *P. aeruginosa* was applied to copper-contaminated soil, the soil pH increased, EC decreased, available copper was reduced, and enzymatic activities rose. Immobilized *P. aeruginosa* also caused changes in the bacterial community structure of the soil, leading to the decrease in alpha diversity and the replacement of the dominant phylum of soil bacteria. Furthermore, immobilized *P. aeruginosa* promoted the growth and nitrogen fixation of Chinese milk vetch planted in copper-contaminated soil, thereby reducing copper accumulation and stress. In summary, biochar-immobilized *P. aeruginosa* can be utilized as an in situ soil amendment and plant growth promoter for the remediation of copper-contaminated soil.

## Figures and Tables

**Table 1:** Alpha diversity indexes.

Treatment	Shannon	Simpson	Evenness	Coverage
CK	9.422 ± 0.060 ^b^	0.007 ± 0.002 ^ab^	0.820 ± 0.004 ^a^	0.980 ± 0.002 ^a^
BC	9.620 ± 0.032 ^a^	0.006 ± 0.001 ^b^	0.829 ± 0.004 ^a^	0.979 ± 0.004 ^a^
FB	9.412 ± 0.124 ^b^	0.006 ± 0.001 ^b^	0.815 ± 0.008 ^ab^	0.978 ± 0.004 ^a^
IM	9.255 ± 0.087 ^c^	0.009 ± 0.001 ^a^	0.802 ± 0.013 ^b^	0.977 ± 0.003 ^a^

Different lowercase letters indicate a significant difference between treatments (*p* < 0.05).

## Data Availability

The original contributions presented in this study are included in the article. Further inquiries can be directed to the corresponding author.
